# Production of Plant-Associated Volatiles by Select Model and Industrially Important *Streptomyces* spp.

**DOI:** 10.3390/microorganisms8111767

**Published:** 2020-11-11

**Authors:** Zhenlong Cheng, Sean McCann, Nicoletta Faraone, Jody-Ann Clarke, E. Abbie Hudson, Kevin Cloonan, N. Kirk Hillier, Kapil Tahlan

**Affiliations:** 1 Department of Biology, Memorial University of Newfoundland, St. John’s, NL A1B 3X9, Canada; zc5251@mun.ca (Z.C.); jodyannc@mun.ca (J.-A.C.); 2 Department of Biology, Acadia University, Wolfville, NS B4P 2R6, Canada; sean.mccann@acadiau.ca (S.M.); elizabeth.martyn@acadiau.ca (E.A.H.); raynecloonan@gmail.com (K.C.); 3 Department of Chemistry, Acadia University, Wolfville, NS B4P 2R6, Canada; nicoletta.faraone@acadiau.ca

**Keywords:** *Streptomyces*, model/industrial species, natural products, VOCs, terpenoids

## Abstract

The *Streptomyces* produce a great diversity of specialized metabolites, including highly volatile compounds with potential biological activities. Volatile organic compounds (VOCs) produced by nine *Streptomyces* spp., some of which are of industrial importance, were collected and identified using gas chromatography–mass spectrometry (GC-MS). Biosynthetic gene clusters (BGCs) present in the genomes of the respective *Streptomyces* spp. were also predicted to match them with the VOCs detected. Overall, 33 specific VOCs were identified, of which the production of 16 has not been previously reported in the *Streptomyces*. Among chemical classes, the most abundant VOCs were terpenes, which is consistent with predicted biosynthetic capabilities. In addition, 27 of the identified VOCs were plant-associated, demonstrating that some *Streptomyces* spp. can also produce such molecules. It is possible that some of the VOCs detected in the current study have roles in the interaction of *Streptomyces* with plants and other higher organisms, which might provide opportunities for their application in agriculture or industry.

## 1. Introduction

Volatile organic compounds (VOCs) are small odorous molecules (up to C20) with low molecular mass (100–500 Daltons), high vapor pressure, low boiling point and a lipophilic moiety [1]. VOCs are biosynthetically produced in plants [2], animals [3], humans [4], fungi [5] and bacteria [6], where they act as information-carrying signals (semiochemicals) between individuals, modulating intra-species or inter-species interactions [7,8,9]. They are grouped into major chemical classes, including hydrocarbons (alkanes, alkenes, alkynes), alcohols, aldehydes, ketones, benzenoids, pyrazines, sulfides and terpenes [6]. Many VOCs are thought to be precursors [10,11,12], intermediates [13] and side products [14,15] of primary or specialized metabolic pathways. It has been reported that the biosynthesis of VOCs commonly occurs using streamlined pathways, sometimes involving a single enzyme [16,17], where mixtures of compounds with similar structures are often produced during the process [14]. VOCs are routinely exploited for their aroma in the food, cosmetic, chemical and pharmaceutical industries [18]. In addition, VOCs have potential uses in agriculture as many exhibit important biological activities, such as insect repellency [19,20], or are used as fungicides [21], bactericides [18,22] and nematicides [23,24]. Chemicals employed in agriculture and other industrial applications are generally manufactured synthetically. However, newer approaches can exploit microbes as biosynthetic sources of relevant compounds [25], providing cheaper, more sustainable and environmentally friendly alternatives for pest management and crop protection.

Non-motile sporulating Gram-positive bacteria from the genus *Streptomyces* are ubiquitous soil dwellers, but are also found in the rhizosphere or as symbionts of plants and invertebrates [26]. The *Streptomyces* are recognized as industrially relevant because of their ability to produce a variety of specialized metabolites, which are widely used in medicine and agriculture [27]. Many members from the genus share similar core genomes, which comprise genes necessary for the normal growth and reproduction of a species [28]. In comparison, they differ from each other in their specialized metabolic capabilities, which often includes groups of genes residing in biosynthetic gene clusters (BGCs) [29]. Specialized metabolism is normally not essential for bacteria, and therefore the genes involved can be gained or lost to the environment more readily due to recombination and horizontal gene transfer [30], leading to diverse production capabilities within a genus.

*Streptomyces* spp. are also known to produce a variety of VOCs, some of which are classified as specialized metabolites [18,31] and have roles in spore dispersal [32], symbiosis [31] or competition [33]. A recently described form of the *Streptomyces* life cycle called exploration is also mediated by the VOC trimethylamine (TMA) [34,35]. When co-cultured with yeast, depletion of glucose in the medium induces TMA production in *Streptomyces venezuelae*, which leads to decreased iron solubility due to pH changes, thereby reducing its availability for other competing microorganisms [34]. In addition, TMA triggers *S. venezuelae* explorative growth, which enables it to rapidly move over solid surfaces [35]. Another example of *Streptomyces* intraspecies signalling involves two common VOCs, namely geosmin and 2-methylisoborneol (2-MIB), which are also known for their strong, earthy odor. The two metabolites attract *Folsomia candida* (springtails), which feed on *Streptomyces* colonies, where spores attach to their hydrophobic cuticles and are dispersed by the soil-dwelling hexapods to new and relatively distant locations [32]. A recent report has also shown that newly mated queens of *Solenopsis invicta* (red imported fire ant) are attracted by geosmin and 2-MIB to *Streptomyces*-rich locations for nesting, which have reduced levels of pathogenic fungi [36]. In addition, 2-MIB attracts *Drosophila melanogaster* (common fruit fly) for oviposition, where other *Streptomyces* specialized metabolites, such as cosmomycin D and avermectin, kill the larvae, providing a potential nutrient source for the *Streptomyces* [37]. Therefore, the *Streptomyces* use different VOCs for eliciting trans-kingdom interactions and for influencing biological outcomes, demonstrating the importance of such molecules in the environment.

In addition to some of the known examples described above, genome mining has revealed an abundance of potential VOC-associated BGCs of unknown function in *Streptomyces* spp., making them good candidates for the production of such compounds [38,39]. Important factors influencing the production of volatiles include media composition, culture conditions, the physiological state of the producer, oxygen availability, moisture, temperature and pH [40]. Therefore, nine *Streptomyces* spp. extensively used as model organisms to study different biological phenomena or in the production of important human and veterinary drugs were chosen for analysis in the current study. The selected microorganisms were cultured under different nutritional conditions and VOC production was analyzed using gas chromatography–mass spectrometry (GC-MS). Using this strategy, we obtained a snapshot of the volatile production capabilities of the respective *Streptomyces* spp., and some of our major findings are discussed below.

## 2. Materials and Methods

### 2.1. Strains and Growth Conditions

Bacteria used for VOC production are listed in Supplemental Table S1. *S. aureofaciens* ATCC 12551 was purchased from Cedarlane Laboratories (Burlington, ON, Canada); *S. coelicolor* M145 [41] was provided by Dr. Mervyn J. Bibb (John Innes Centre, Norwich, UK). All other species (*S. avermitilis*, *S. clavuligerus*, *S. exfoliatus* SMF19, *S. griseofuscus*, *S. hygroscopicus* NRRL 15879, *S. lividans* TK24 and *S. parvulus* B1682) were gifts from Dr. Susan E. Jensen (University of Alberta, Edmonton, AB, Canada). All media/reagents were purchased from Fisher Scientific (Mississauga, ON, Canada) or VWR International (Mississauga, ON, Canada). All *Streptomyces* cultures were grown at 28 °C and liquid cultures were agitated by shaking at 200 rpm. Spore stocks were prepared using International *Streptomyces* Project (ISP)-4 medium; seed cultures were grown using 5 mL of BD™ Tryptic Soy Broth (TSB) for 48 h. For inoculation, 1% *v*/*v* of seed culture was transferred to 25 mL of YMS [42], SFM [43] or synthetic [44] medium in 125 mL flasks, respectively, which were then incubated for five days.

### 2.2. Volatile Organic Compound Collection and Sampling

For VOC analyses, cultures of each strain grown in the three growth media (25 mL each, 75 mL in total) were mixed together, and 25 mL of this mixture was transferred into one 8 oz Mason/Canning jar (Bernardin Ltd., Brampton, ON, Canada). As a control, volatiles were also collected from uninoculated media processed similarly. Volatile collection and extraction was performed according to the method described by Light et al. [45] with some modification. Jar lids were pierced to make ports in order to attach tubing for the push-pull volatile collection system. Lids were sealed and connected with polypropylene tubing to a PVAS22 pump system (Portable Volatile Assay System; Rensselaer, NY, USA). Pre-cleaned divinylbenzene polymer volatile traps (HayeSep-Q 80/100 mesh, 27+ mg; Volatile Assay Systems, Rensselaer, NY, USA) were used for volatile collection. Traps were pre-cleaned using established protocols [45], flushing sequentially with methanol, acetone and HPLC-grade hexane. A continuous flow of carbon-filtered air was passed through each container (0.3 L/min push and 0.1L/min pull), with polymer traps placed on the pull line to collect volatiles emitted from cultures. Volatiles were collected for 12 h at room temperature, following which traps were wrapped in aluminum foil and stored at −20 °C until elution with solvent. After volatile collection, equipment was cleaned with 95% ethanol followed by distilled water and dried at room temperature.

### 2.3. Volatile Organic Compound Analysis and Identification

Samples were analyzed using gas chromatography–mass spectrometry (GC-MS) to determine the composition of VOCs being released from control (sterile medium) and media inoculated with different *Streptomyces* spp. Volatiles collected using the PVAS22 system were eluted in 200 μL of hexane (CHROMASOLV, >98.5% purity, Sigma-Aldrich, Saint Louis, MO, USA) under a flow of nitrogen. Extracts were concentrated under ultra-high purity nitrogen to 75 μL and transferred to a 250 μL glass insert. Samples were analyzed using a Scion 456 Gas Chromatograph–Single Quad Mass Spectrometer (GC-MS; SCION Instruments, Livingston, UK). A non-polar capillary column Rxi^®^-5silms (30 m × 0.25 mm, film thickness 0.25 mm; Restek Corporation, State College, PA, USA) linked to a Bruker mass spectrometer (Bruker Daltonics Ltd., Coventry, UK) was used for analysis. Samples underwent splitless injection. The oven temperature was programmed for 1 min at 40 °C then increased to 100 °C at 6 °C/min and held for 1 min, then increased to 250 °C at 10 °C/min, and temperature held for 5 min. Both the injector and transfer line were maintained at 250 °C. Helium was used as a carrier gas. We used a detection threshold of 100 MCPS (megaCounts per second) to assemble a list of the high-abundance volatiles in each sample. These compounds were identified using analytical standards and NIST Mass Spectral Search Program for the NIST/EPA/NIH Mass Spectral Library Version 2.0 g build in 2011 (Scion Instruments UK Ltd., Livingston, West Lothian, UK, hereafter “NMSS”). Matches suggested by NMSS were evaluated based on spectral similarity, as well as comparison of their Kovats retention indices (RI) to published values (Supplemental Table S2). Identifications were primarily based on the availability of spectra in NMSS, and the stereochemistry which we have assigned should be interpreted with caution. Uninoculated medium was also subject to the same volatile trapping, sampling and analysis to serve as a control. Compounds from the bacterial samples that were also detected in control samples were considered media-derived or artifacts of the collection and sampling procedure, and not of bacterial origin. We were unable to identify all compounds present in the samples, but compounds with good spectral matches, and subsequent injection of authentic standards, or supported by published RI data (absolute difference ≤ 10), were considered as high confidence matches. If no authentic standard was available, and the RI could not support the NMSS suggested compound ID, we considered the match to have low confidence. We further classified the identified compounds by functional group and putative origin (bacterial or medium-derived).

### 2.4. Streptomyces Phylogenetic and BGC Analysis

Strains for which genomic data were used for phylogenetic and BGC analysis are listed in Supplemental Table S1. To build a concatenated gene-based tree, the DNA sequences of five housekeeping genes, *atpD*, *gyrB*, *recA*, *rpoB* and *trpB*, were downloaded and aligned with MAFFT (https://mafft.cbrc.jp/alignment/server/). The evolutionary history was inferred by using the maximum likelihood method and the general time reversible model, and the tree with the highest log likelihood (−39385.69) is shown. Initial tree(s) for the heuristic search were obtained automatically by applying Neighbor-Join and BioNJ algorithms to a matrix of pairwise distances estimated using the Maximum Composite Likelihood (MCL) approach and then selecting the topology with superior log likelihood value. This analysis involved ten nucleotide sequences and there were 10,954 positions in the final dataset. Evolutionary analyses were conducted in MEGA X [46] using a bootstrap value of 100. *Mycobacterium tuberculosis* H37Rv (NCBI accession number NC_000962.3) served as an out-group.

BGC prediction was performed using the bacterial version of antiSMASH 5.0 [38].

## 3. Results and Discussion

Nine *Streptomyces* spp. used as model organisms to study various aspects of general biology (*S. coelicolor* and *S. lividans*), protein and specialized metabolite production (*S. exfoliatus*, *S. griseofuscus*, *S. hygroscopicus* and *S. parvulus*), or large scale industrial production of pharmaceutically important compounds (*S. aureofaciens*: producer of tetracyclines, *S. avermitilis*: producer of avermectins and *S. clavuligerus*: producer of clavulanic acid), were chosen for analysis in the current study. Prior to culturing the respective species for VOC detection, their publicly available genome sequences were examined to obtain a better understanding of their relative phylogeny and genetic capabilities for specialized metabolism.

### 3.1. Relative Phylogeny and Biosynthetic Potential of Select Streptomyces spp.

**Figure 1:**
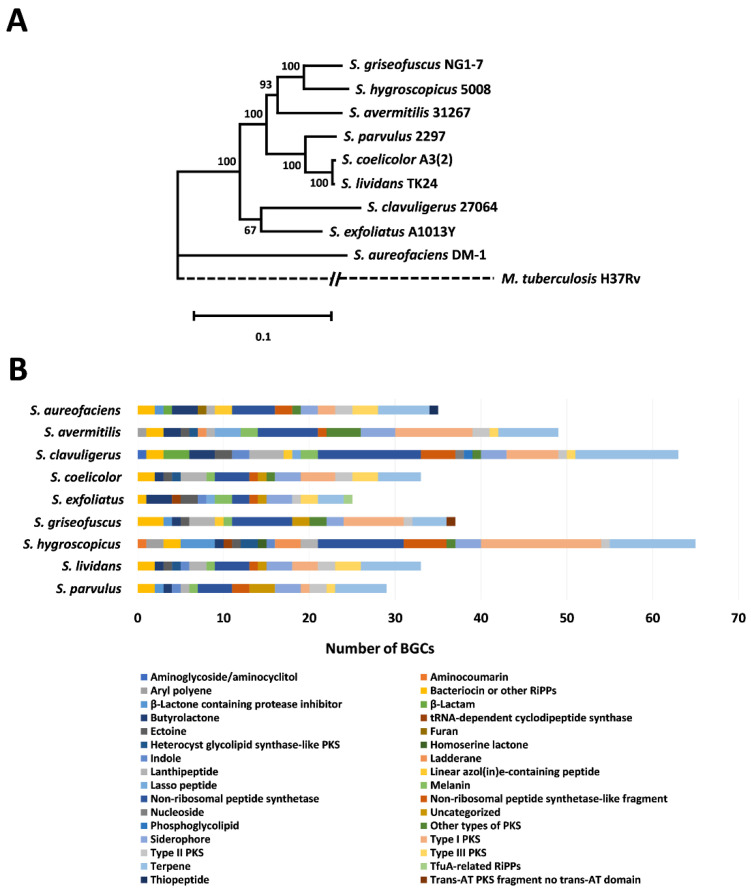
Relative phylogeny and BGC content of *Streptomyces* spp. used in the current study. (**A**) Maximum likelihood tree of the respective *Streptomyces* spp. based on five concatenated housekeeping genes: *atpD*, *gyrB*, *recA*, *rpoB* and *trpD*. Bootstrap values were calculated using 100 replicates and *Mycobacterium tuberculosis* served as an outgroup. The percentage of trees in which the species clustered together is shown next to the branches. The tree is drawn to scale, with branch lengths measured in the number of substitutions per site. (**B**) Predicted BGC numbers and their respective classes (listed below) as per antiSMASH.

The genome sequences of the nine *Streptomyces* spp. chosen in this study were obtained from the National Center for Biotechnology Information (NCBI) database. The sequences were selected based on quality and completeness, and the genomes ranged in size from 6.51 to 10.38 Mbp (Supplemental Table S1). The phylogenetic relationship between the different *Streptomyces* spp. was analyzed by constructing a concatenated tree using the DNA sequences of five housekeeping genes: *atpD*, *gyrB*, *recA*, *rpoB* and *trpB* [29,47,48,49,50,51,52]. To obtain a genetic overview of their specialized metabolic capabilities, BGCs were predicted using antiSMASH [38]. The resulting phylogenetic tree showed that the selected organisms distributed into two clades, and one distinct branch formed by *S. aureofaciens* DM‑1 (Figure 1A). In addition, three species (*S. hygroscopicus* 5008, *S. avermitilis* 31267 and *S. griseofuscus* NG1-7) clustered together and contained the highest numbers of BGCs on their chromosomes (Supplemental Table S1). Of the selected organisms, *S. aureofaciens* DM-1, *S. avermitilis* MA-4680, *S. clavuligerus* ATCC 27064, *S. parvulus* 2297, *S. coelicolor* A3(2) and *S. hygroscopicus* 5008 are also known to contain plasmids. In addition, plasmids from three species contain predicted BGCs, with the highest number being 30 in the case of *S. clavuligerus* ATCC 27064 (Supplemental Table S1). *S. coelicolor* A3(2) also contains two plasmids, one of which harbors three predicted BGCs. However, because the *S. coelicolor* strain (M145) used in the current study lacks plasmids [41], we did not include their sequences during analysis.

The examined genomes contain a variety of BGCs belonging to the major biosynthetic classes (Figure 1B and Supplemental Table S3). In total, 369 BGCs from 32 categories were detected in the nine *Streptomyces* spp. The three most abundant categories were those containing terpene synthases (60/16.3%), non-ribosomal peptide synthetases (NRPSs, 55/14.9%) and type I polyketide synthases (T1PKSs, 46/12.5%) (Supplemental Table S3). This is consistent with previous reports where terpene synthases were shown to be widely distributed in different *Streptomyces* [53,54,55]. Each *Streptomyces* genome contained between 25 and 65 BGCs (Supplemental Tables S1 and S3), where *S. hygroscopicus* contained the highest number (*n* = 65), which is consistent with it having the largest genome (10.38 Mbp) in the current study. Despite its relatively small chromosome, *S. clavuligerus* contained the second highest number of BGCs (*n* = 63), due to the 30 that are located on the 1.8 Mbp plasmid present in this species. In comparison, *S. exfoliatus* had the lowest number of BGCs (*n* = 25) amongst the species analyzed in the current study (Supplemental Tables S1 and S3). Overall, there was no correlation between the concatenated tree-based phylogeny and specialized metabolite BGC content (Figure 1). This is consistent with results from previous studies which showed that specialized metabolite BGCs are highly variable and only a few of them are conserved among different *Streptomyces* spp. [56]. The highly variable distribution of BGCs in *Streptomyces* genomes is thought to be the result of gene loss and horizontal gene transfer, which allows the host bacteria to gain or lose BGCs to cope with selective pressures [57]. Therefore, we proceeded to analyze VOC production in the nine *Streptomyces* spp. selected, and BGC predictions were only used to guide VOC identification when possible.

### 3.2. Overview of VOC Production by Select Streptomyces spp.

**Figure 2:**
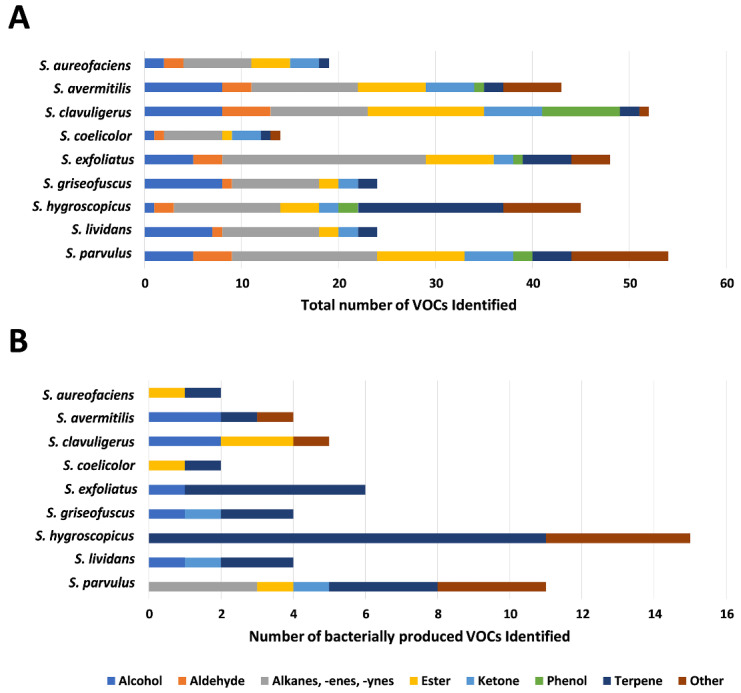
Identified volatile organic compounds (VOC) grouped according to functional classes. (**A**) Total identified VOCs attributed to bacterial origin and those derived from the media. (**B**) Identified VOCs of bacterial origin only.

We identified 128 unique VOCs from the headspace of *Streptomyces* cultures grown under three different nutritional conditions (Figure 2A, Supplemental Table S4). After accounting for volatile compounds present in the control samples (blank media and experimental apparatus), we found 33 unique chemical entities that we attribute to bacterial origin (Figure 2B, Supplemental Table S5). Peak-by-peak examination of chromatograms revealed that these numbers underestimate the total volatile species detected, as many of them could not be matched to standards (Supplemental Table S4); henceforth, we only discuss the high abundance peaks that were assigned identities in our study. The most abundant VOC BGCs predicted in the respective *Streptomyces* spp. from the current study were associated with terpenes (Figure 1B, Supplemental Table S3), and our results showed that bacterially derived compounds, both in quantity and relative abundance, were dominated by members from this class (Table 1). However, it should be noted that there was substantial variation in the distribution of molecular types of terpenes and other VOCs produced across different species (Figure 2B, Table 1).

The largest number of *Streptomyces*-specific VOCs identified (*n* = 15, of which 11 were terpenes) were from *S. hygroscopicus* (Figure 2B, Supplemental Table S5), which also contains the highest number of terpene-associated BGCs (*n* = 10) (Figure 1B, Supplemental Table S3). However, we did not observe a similar trend in all species analyzed as in some cases the number of predicted VOC BGCs did not correlate with those actually detected. For instance, *S. clavuligerus*, which is known to produce a wide range of specialized metabolites, including β-lactam antibiotics and molecules of plant origin [114], contains many predicted BGCs (Supplemental Table S3) and produced five identifiable VOCs (Figure 2B, Supplemental Table S5). However, terpenes were not detected in *S. clavuligerus* headspace samples in our study (Figure 2B, Supplemental Table S5), even though the species possesses 12 predicted BGCs for such metabolites (Figure 1B, Supplemental Table S3). Previous reports have shown that the plant-associated terpenoids carveol, cuminyl alcohol and hydroxyvalerenic acid were detected during liquid chromatography–MS/MS analysis of certain *S. clavuligerus* cultures extracted with methanol/ethyl acetate [114]. In a separate report, terpenes were not detected in *n*-hexane extracts of *S. clavuligerus* cultures grown on SFM or YMS media (also used in the current study) when subjected to GC-MS analysis [53]. It is possible that many of the terpenes produced by such species might not have matching standards for identification, or could be soluble metabolites instead of VOCs, which was the focus of the current study. Therefore, further optimization of culture media/conditions and detection methods might be required to explore the VOC production potential of certain *Streptomyces* spp.

### 3.3. Identification of Previously Reported Streptomyces-Specific VOCs

**Figure 3:**
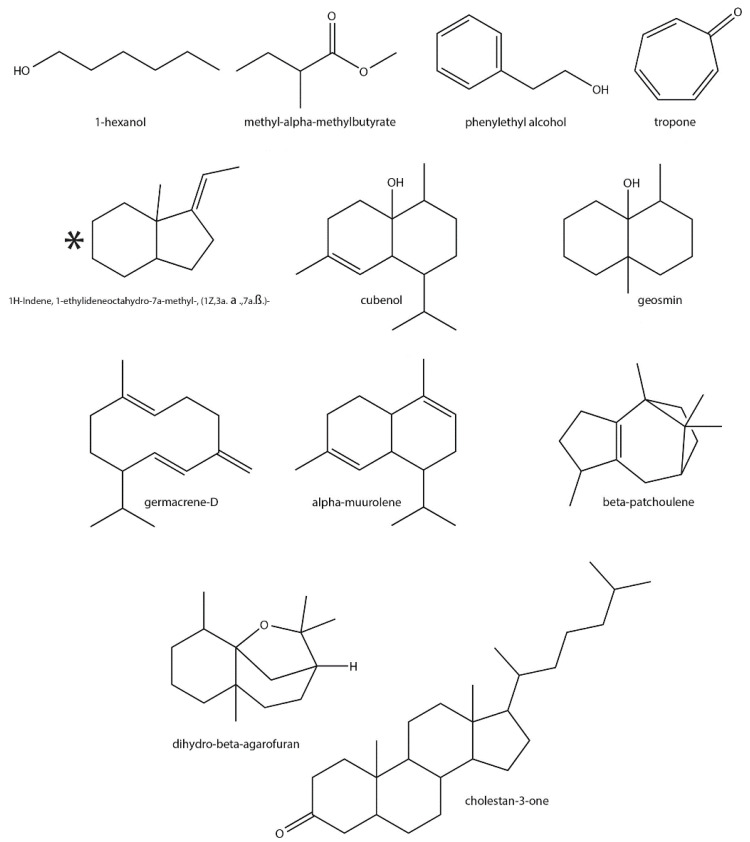
Structures of plant-associated VOCs detected in the current study that have been previously reported from *Streptomyces* spp. The asterisk (*) indicates that the same spectrum was found in two well-separated peaks, indicating the likely occurrence of two closely related diastereomers.

Thirty-three *Streptomyces*-specific VOCs were identified in the current study, of which 17 (including nine terpenoids) have been reported to be produced by members of the genus previously (Figure 3, Table 1). Geosmin and 2-MIB were the most common volatile specialized metabolites detected. The production of geosmin has also been reported in other microorganisms, as well as in plants [115]. Due to its inhibition of chemotaxis, oviposition and feeding in *Drosophila* spp. [116], geosmin has been considered as a potential insect repellent for use in agriculture. Germacrene-D, which was also detected during the analysis, is an intermediate of the geosmin biosynthetic pathway [96], whereas 2-MIB production is widely distributed in bacteria including the *Streptomyces* [53]. Moreover, 2-MIB also shows antifungal activity against *Fusarium moniliforme* (Family: Nectriaceae), which is known to cause cutaneous disease in humans [117]. Additionally, 2-Methyl-2-bornene is an isomeric homo-monoterpene of 2-MIB produced using same pathway [73,74], which has been reported in some species of polydesmid millipede (arthropods) [118] and *Sclerotinia sclerotiorum* (plant pathogenic fungus) [119].

Other terpenoids detected in the current study include α-muurolene (Figure 3), which is also produced by *S. globisporus* [76]. In addition, α-muurolene is a component of the phytotoxic and antimicrobial essential oils derived from the plants *Argemone ochroleuca* (Mexican poppy) [120] and *Carum montanum* (Family: Apiaceae) [121], respectively. Cubenol has also been previously detected in the headspace of *S. griseus* DSM40236 cultures [87]. Dihydro-β-Agarofuran has been reported in *S. albus* [94] and in multiple plants, and has been shown to possess antitumor, immunosuppressant, antiviral and insecticidal activities [95]. Cholestan-3-one is a cholesterol oxidation product from a marine *Streptomyces* spp. [104] and was also detected in the current analysis (Table 1). Whereas β-patchoulene is also produced by *S. albus* [94], 1H-indene, 1-ethylideneoctahydro-7a-methyl-, (1Z,3a.α.,7a. β.)- by *S. alboflavus* [99] and 1H-indene, 1-ethylideneoctahydro-7a-methyl-, cis- by *S. globisporus* [76], but no specific functions or activities have been attributed to the respective VOCs.

Organic alcohols were another major class of VOCs detected in the current study (Figure 3, Table 1). For example, 1-hexanol is also produced by plants, fungi and certain bacteria, including *S. albidoflavus* [122]. It constitutes the odor of some fruit varieties [60,61,62], and when combined with other molecules, 1-hexanol has been reported to have insect repellent properties due interference with the host-seeking mechanism in mosquitoes [123]. On the other hand, *Scolytus schevyrewi* (banded elm bark beetles) are attracted by 1-hexanol production in apricot trees, indicating a role for it in host tree selection [124]. Another detected compound was phenylethyl alcohol (Table 1), an aromatic alcohol produced by some *Streptomyces* spp. [18,63,64,125] and by plants such as rose [65]. It has been shown to function as an insect repellent against *Rhodnius prolixus* (Family: Reduviidae) and *Triatoma infestans* (kissing bug), both of which are vectors of the Chagas disease parasite [77]. Phenylethyl alcohol also displays antifungal activity against the plant pathogen, *Ceratocystis fimbriata* (Family: Ceratocystidaceae) [63]. Examples of other previously reported VOCs detected in the current study include tropone, a non-benzenoid aromatic compound with antibiotic activity produced by bacteria, including the *Streptomyces* [70,126,127]. Methyl α-methylbutyrate is also an antimicrobial produced by *Streptomyces yanglinensis* [64] and by plants, including wild strawberries [109], *Chanmemelum nobil* (Roman chamomile) [110] and *Malus domestica* (apple) [111]. Other detected compounds included 2,2,3,3-tetramethyl-cyclopropanecarboxylic acid, 1-butylhexyl ester and 6-Methyl-cyclodec-5-enol, which have not been previously identified in *Streptomyces* spp. However, molecules with similar functional groups and structures have been described from the genus, suggesting that the *Streptomyces* are capable of producing such metabolites [106].

It is worth noting that among the 17 known VOCs previously reported from *Streptomyces*, 13 are also produced by plants (Figure 3, Table 1). Although 1H-indene, 1-ethylideneoctahydro-7a-methyl-, (1Z,3a. α.,7a.β.)- and 1H-indene, 1-ethylideneoctahydro-7a-methyl-, cis- have not been reported in plants, a molecule with similar structure (1H-indene, 1-ethylideneoctahydro-7a-methyl-, (1E, 3a. α, 7a.β.)) is synthesized by *Brickellia cavanillesii* (Family: Asteraceacae) [100]. In addition, 2-MIB and 2-methyl-2-bornene are not found in plants, but they have been detected in algae [128] and in the millipede *Niponia nodulosa* (Family: Cryptodesmidae) [118,129]. *Streptomyces* spp. are known plant symbionts, and they have been identified in various arthropod species found to produce specialized metabolites [26]. Therefore, our results suggest that the presence of *Streptomyces* spp. and their possible contribution to specialized metabolite/VOC production in plants and arthropods should be taken into consideration while studying such phenomena in the future.

### 3.4. Identification of VOCs Previously Unreported in Streptomyces spp.

**Figure 4:**
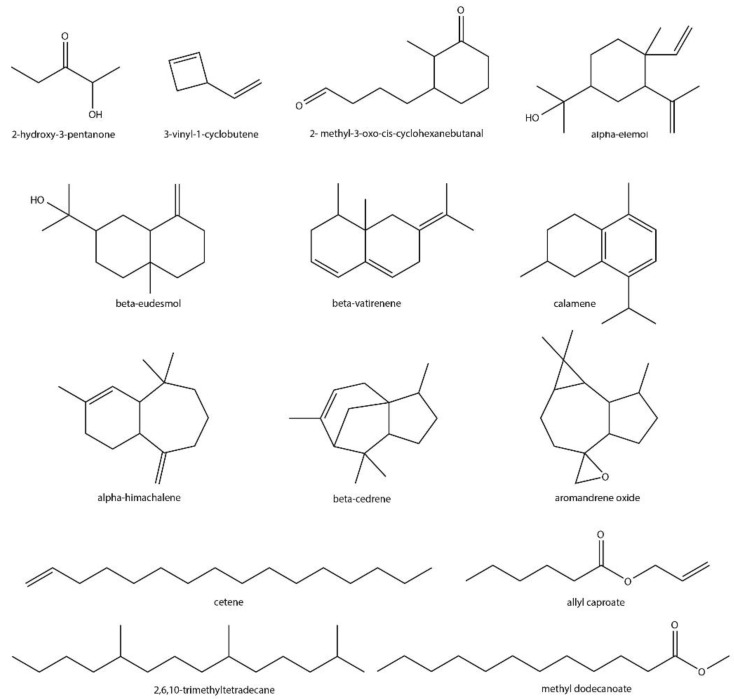
Structures of plant-associated VOCs detected in the current study that have not been previously reported from *Streptomyces* spp.

Sixteen VOCs identified in the current study have not been previously reported from *Streptomyces*, and seven of them are terpenoids (Figure 4, Table 1). In addition, the majority (14 out of 16) of these VOCs were also found to be plant-associated (Figure 4). α-Elemol is a terpene alcohol produced by *Calendula officinalis* (pot marigold) with applications in phytotherapy for skin inflammation, but it has been shown to have some in vitro cellular toxicity [75]. α-Himachalene is an insect pheromone of *Phyllotreta* and *Aphthona* flea beetles [130,131], but it is also produced by certain plants and has some insecticidal [78], antimicrobial [79] and antipsoriatic [80] activities. In addition, α-himachalene is a major constituent in Atlas cedarwood oil, which is used in drug and perfume formulations [132]. Other detected compounds were β-eudesmol, β-cedrene, β-vatirenene, aromadendrene oxide-(2) and calamene (Table 1). β-Eudesmol is produced by plants such as *Atractylodes lancea* (Family: Asteraceae) [84] and *Zingiber zerumbet* (bitter ginger) [85], and has a wide range of bioactivities including antiangiogenicity [84], inhibition of Na^+^, K^+^-ATPase activity [133] and stimulation of feeding behavior in rats [134]. β-Cedrene is a component of *Juniperus virginiana* (eastern red cedar) essential oil [90] and the floral scent of Bearded Irises (Family: Iridaceae) [91]. β-Vatirenene has antioxidant activity and is produced by the plants *Valeriana jatamansi* (Family: Valerianaceae) [92] and *Parthenium hysterophorus* (Santa Maria feverfew) [93]. Aromadendrene oxide-(2) is a component of *Pamburus missionis* (Family: Rutaceae) essential oil [102], which induces apoptosis in skin epidermoid cancer cells [135,136], whereas calamene is extracted from *Eryngium carlinae* (toad’s herb) and displays potential antioxidant activity [103].

Nine *Streptomyces*-specific VOCs found in this study were non-terpenoid molecules (Figure 4). p-Cresol (an alcohol) is produced from tyrosine [137,138] by 4-hydroxyphenylacetate decarboxylases [139]. It increases anxiety-like behaviors in mice [140], has cell toxicity [141] and acts as an insect attractant [142]. Moreover, 3-vinyl-1-cyclobutene is an unsaturated cyclic hydrocarbon found in the extract of *Cornus officinalis* (Japanese cornel), a traditional Chinese herbal medicine [66]. Cetene (a hydrocarbon) is normally found in *Tinospora cordifolia* (heart-leaved moonseed) [67] and *Lathyrus sativus* (grass pea) [68], where female *Aphis craccivora* (cowpea aphids) are attracted by it in *Lathyrus sativus* [68]. Moreover, 2,6,10-trimethyltetradecane (another hydrocarbon) is produced by *Cyrtocarpa procera* (Chupandia plant) and has been shown to be toxic to *Artemia salina* (brine shrimp) [69]. Another identified compound was 2-hydroxy-3-pentanone (a ketone), which is known to be synthesized by a wide range of organisms, including bacteria [143,144,145], yeast [146], human epithelial cells [147] and during the roasting of coffee beans [72]. In addition to the major groups described above, four additional VOCs from other chemical classes were also identified in our analysis. Allyl caproate, an unsaturated ester, is found at low levels in fruits [148], mushrooms [149] and leaf oils [150]. It mimics the flavor of pineapples and is therefore used as a food additive [107]. Methyl dodecanoate is a potential insect repellent with activity against *Aedes aegypti* (yellow fever mosquito) [112]. It is produced by common aquatic duckweed species, *Spirodela polyrrhiza* (HZ1) and *Lemna minor* (WX3), and has also been shown to stimulate nitrogen utilization by bacteria for remediation purposes [113]. Cyclohexanebutanal, 2-methyl-3-oxo-, cis- and isobutyl tetradecyl carbonate were also detected for the first time in the *Streptomyces*, but we were unable to find any information on their biosynthesis in other organisms or their biological activities, respectively.

## 4. Conclusions

Certain *Streptomyces* spp. are known to produce VOCs that are normally associated with higher organisms [18]. Many VOCs produced by plants have extensive applications in agriculture, medicine, food and cosmetic industries [151], but their large scale production can be limiting due to low titers or availability of the natural host [152]. In addition, the chemical synthesis of such compounds at industrial levels may also represent a challenge [153]. The finding that some *Streptomyces* spp. can naturally synthesize certain plant-associated VOCs further highlights the possibility of using these bacteria for the large-scale production of such molecules. As prokaryotes, *Streptomyces* are relatively easy to manipulate and culture, allowing for the improved and sustainable production of certain chemicals [154]. For example, a terpenoid-production platform was recently developed for *Streptomyces reveromyceticus* and the titer of botryococcene (a triterpene) was increased by manipulating biosynthetic genes and promoters [155]. It has been reported that VOC biosynthesis is commonly catalyzed by products of single genes rather than gene clusters [16,17,156,157], further simplifying the complexity of gene manipulations required for improving yields.

The ecological function of many plant VOCs, such as those produced by flowering varieties to attract pollinators, is well-documented [158]. As a defense mechanism, toxic or repellent VOCs produced by plants also deter attacking herbivores, while other VOCs can mediate plant–plant interactions to coordinate growth and protective responses, respectively. In addition, the antimicrobial activities of VOCs are known to directly empower plants against invading pathogens. Intriguingly, some VOCs produced by plant-associated bacteria, including *Streptomyces*, also have similar functions [159]. For example, 3-octanone produced by *S. coelicolor*, promotes the growth of *Arabidopsis thaliana* by altering auxin/cytokinin homeostasis [160]. Other VOCs produced by *Streptomyces albulus* inhibit the growth of the fungal pathogens *Sclerotinia sclerotiorum* (Family: Sclerotiniaceae) and *Fusarium oxysporum* (Family: Nectriaceae), which are responsible for cucumber Fusarium wilt and sclerotinia stem rot of oilseed rape, respectively [161]. In addition, inhibition of the feeding behavior of *D. melanogaster* by geosmin produced by the *Streptomyces* has already been discussed in detail [116]. Although the production of some plant-associated VOCs in the *Streptomyces* has been previously reported [114], we used three types of growth media and identified 27 such metabolites from cultures of just nine species (Figure 3 and Figure 4, Table 1). It should also be noted that our study detected such metabolites under laboratory conditions and that their production in the natural environment by some *Streptomyces* spp. needs to be verified. Understanding the ecological roles of such VOCs can allow for their exploitation in different fields, particularly in agriculture, where microbial products can provide greener alternatives for managing plant diseases or to promote growth [162]. Furthermore, some of the discussed *Streptomyces* VOCs also have applications in the food, cosmetic, chemical and pharmaceutical industries. Therefore, with the increasing number of new metabolites being routinely identified from different *Streptomyces* spp., members from the genus can also serve as an important resource for the industrial production of both endogenous and exogenous VOCs for future use.

## Figures and Tables

**Table 1:** *Streptomyces*-specific VOCs identified in the current study.

Chemical Class	VOC ^¥^	Functional Group	ID ^ψ^	Detected in ^ϕ^	Previously Reported in *Streptomyces*	Previously Reported in Plants
**Alcohol**	1-Hexanol	Alkane, alcohol	**	*S. ave, S. gri, S. liv*	Yes [18,58,59]	Yes [60,61,62]
	p-Cresol	Aromatic alcohol	*	*S. clav*	No	No
	Phenylethyl alcohol	Aromatic alcohol	***	*S. ave, S. exf*	Yes [63,64]	Yes [65]
**Hydrocarbon**	3-Vinyl-1-cyclobutene	Alkene	*	*S. par*	No	Yes [66]
	Cetene	Alkene	*	*S. cla, S. par*	No	Yes [67,68]
	2,6,10-trimethyltetradecane	Alkane	*	*S. par*	No	Yes [69]
**Ketone**	Tropone	Aromatic ketone	*	*S. par*	Yes [70]	Yes [71]
	2-Hydroxy-3-pentanone	Alcohol, ketone	**	*S. gri, S. liv*	No	Yes [72]
**Terpenoids**	2-Methyl-2-bornene	Irregular monoterpene	***	*S. exf*	Yes [73,74]	No
	α-Elemol	Sesquiterpene alcohol	*	*S. par*	No	Yes [75]
	α-Muurolene	Sesquiterpene	**	*S. aur*	Yes [76]	Yes [63,77]
	α-Himachalene	Sesquiterpene	*	*S. cla*	No	Yes [78,79,80]
	2-MIB	Monoterpene alcohol	***	*S. exf, S. gri, S. liv, S. par*	Yes [54,81,82,83]	No
	β-Eudesmol	Sesquiterpene alcohol	**	*S. exf, S. hyg*	No	Yes [84,85]
	Geosmin	Irregular sesquiterpene alcohol	***	*S. ave, S. coe, S. exf, S. gri, S. hyg, S. liv, S. par*	Yes [53]	Yes [86]
	Cubenol	Sesquiterpene alcohol	**	*S. exf*	Yes [87]	Yes [88,89]
	β-Cedrene	Sesquiterpene	*	*S. hyg*	No	Yes [90,91]
	β-Vatirenene	Sesquiterpene	*	*S. hyg*	No	Yes [92,93]
	dihydro- β-Agarofuran	Sesquiterpene lactone	*	*S. hyg*	Yes [94]	Yes [95]
	Germacrene-D	Sesquiterpene	**	*S. hyg*	Yes [96]	Yes [97,98]
	1H-Indene, 1-ethylideneoctahydro-7a-methyl-, (1Z, 3a. α.,7a.β.)-	Irregular sesquiterpene	*	*S. hyg*	Yes [99]	Yes [100] ^†^
	1H-Indene, 1-ethylideneoctahydro-7a-methyl-, cis-	Irregular sesquiterpene	*	*S. hyg*	Yes [76]	Yes [100] ^†^
	β-Patchoulene	Sesquiterpene	*	*S. hyg*	Yes [94]	Yes [101]
	Aromadendrene oxide-(2)	Sesquiterpene oxide	*	*S. hyg*	No	Yes [102]
	Calamene	Aromatic sesquiterpene	*	*S. hyg*	No	Yes [103]
	Cholestan-3-one	Triterpenoid ketone	*	*S. hyg*	Yes [104]	Yes [105]
**Other**	2,2,3,3-Tetramethyl-cyclopropanecarboxylic acid, 1-butylhexyl ester	Ester, carboxylic acid	*	*S. par*	Yes [106] ^‡^	No
	Allyl caproate	Ester, alkene	*	*S. hyg*	No	Yes [107]
	Cyclohexanebutanal, 2-methyl-3-oxo-, cis-	Ketone, aldehyde	*	*S. hyg*	No	Yes [108]
	Methyl α-methylbutyrate	Ester	*	*S. coe*	Yes [64]	Yes [109,110,111]
	Methyl dodecanoate	Ester	**	*S. ave*	No	Yes [112,113]
	6-Methyl-cyclodec-5-enol	Enol	*	*S. hyg*	Yes	No
	Isobutyl tetradecyl carbonate	Ester, alkane	*	*S. par*	No	No

Notes: ^¥^ Stereochemical assignments are tentative due to the detection method used. ^ψ^ Identification (ID) based on spectrum, retention index (RI) and injection of authentic standards (***); on spectrum and RI (**); or on NIST spectral match only (*). **^ϕ^** Organisms are represented by first three letters of species names. ^†^ Similar in structure/function to known VOCs produced by plants. ^‡^ Similar in structure/function to known VOCs produced by *Streptomyces* spp.

## Supplementary Materials

The following are available online at https://www.mdpi.com/2076-2607/8/11/1767/s1. Table S1: Details of *Streptomyces* spp. used in the current study and their genome sequences. Table S2. GC/MS data for chemical species derived from select *Streptomyces* spp. grown in liquid culture. Table S3: Numbers of BGCs grouped by product class present in *Streptomyces* spp. used in the current study. Table S4. Total number of VOCs from different chemical classes identified. Table S5. Numbers of *Streptomyces* specific VOCs from different chemical classes identified.
