# Supplementary material for: Production of Plant-Associated Volatiles by Select Model and Industrially Important Streptomyces spp
Source: Microorganisms. 2020 Nov 11;8(11):1767. doi: 10.3390/microorganisms8111767 (PMC7697265; doi:10.3390/microorganisms8111767)
Supplement: Supplementary file 1 [file microorganisms-08-01767-s001.zip › Cheng_McCann_Supplemental_Tables_S1,S3,S4,S5_2020.docx]

**Supplemental Table S1.** **Details of *Streptomyces* spp. used in the current study and their genome sequences.**

| Species | Strain * | Accession number of chromosome/size/BGCs ^ψ^ | Accession number of plasmid/size/BGCs ^ψ^ | Total BGCs ^ψ^ |
| --- | --- | --- | --- | --- |
| *S. aureofaciens* | DM-1 | NZ_CP020567.1/6.82/35 | NZ_CP020568.1/0.25/0 | 35 |
| *S. avermitilis* | MA-4680 | NC_003155.5/9.03/49 | NC_004719.1/0.09/0 | 49 |
| *S. clavuligerus* | ATCC 27064 | NZ_CP027858.1/6.75/33 | NZ_CP027859.1/1.80/30 | 63 |
| *S. coelicolor* | A3(2) strain:CFB_NBC_0001 | NZ_CP042324.1/8.67/33 | SCP1: AL589148.1/0.36/3  SCP2: NC_003904.1/0.03/0 | 36 |
| *S. exfoliatus* | A1013Y | NZ_CP040244.1/7.65/25 | N/A | 25 |
| *S. griseofuscus* | NG1-7 (scaffold) | NZ_MTKC01000003.1/6.51/37 | N/A | 37 |
| *S. hygroscopicus* | 5008 | NC_017765.1/10.15/65 | pSHJG1: NC_017766.1/0.16/0  pSHJG2: NC_016972.1/0.07/0 | 65 |
| *S. lividans* | TK24 | NZ_CP009124.1/8.35/33 | N/A | 33 |
| *S. parvulus* | 2297 | NZ_CP015866.1/7.15/22 | pSPA1: NZ_CP015867.1/0.62/7 | 29 |

* The most complete sequence of the best-characterized strain from the NCBI genome database (ncbi.nlm.nih.gov) was select for BGC content analysis.

^ψ^  Biosynthetic gene clusters (BGCs) were predicted using the online antiSMASH web server (antismash.secondarymetabolites.org).

N/A: not applicable.

**Supplemental Table S3.** **Numbers of BGCs grouped by product class present in *Streptomyces* spp. used in the current study.**

| BGC* | *S. aureofaciens* DM-1 | | *S. avermitilis* ATCC 31267 | *S. clavuligerus* ATCC 27064 | *S. coelicolor* A3(2) | *S. exfoliatus* A1013Y | *S. griseofuscus* NG1-7 | *S. hygroscopicus* 5008/TL101 | *S. lividans* TK24 | *S. parvulus* 2297 | Total |
| --- | --- | --- | --- | --- | --- | --- | --- | --- | --- | --- | --- |
| Aminoglycoside/aminocyclitol | 0 | 0 | | 1 | 0 | 0 | 0 | 0 | 0 | 0 | **1** |
| Aminocoumarin | 0 | 0 | | 0 | 0 | 0 | 0 | 1 | 0 | 0 | **1** |
| Aryl polyene | 0 | 1 | | 0 | 0 | 0 | 0 | 2 | 0 | 0 | **3** |
| Ribosomally synthesised and post-translationally modified peptides (RiPPs) | 2 | 2 | | 2 | 2 | 1 | 3 | 2 | 2 | 2 | **18** |
| β-Lactone containing protease inhibitor | 1 | 0 | | 0 | 0 | 0 | 1 | 4 | 0 | 1 | **7** |
| β-Lactam | 1 | 0 | | 3 | 0 | 0 | 0 | 0 | 0 | 0 | **4** |
| Butyrolactone | 3 | 2 | | 3 | 1 | 3 | 1 | 1 | 1 | 1 | **16** |
| tRNA-dependent cyclodipeptide synthase | 0 | 0 | | 0 | 0 | 1 | 0 | 1 | 0 | 0 | **2** |
| Ectoine | 0 | 1 | | 2 | 1 | 2 | 1 | 1 | 1 | 0 | **9** |
| Furan | 1 | 0 | | 0 | 0 | 0 | 0 | 0 | 0 | 0 | **1** |
| Heterocyst glycolipid synthase-like PKS | 0 | 1 | | 0 | 1 | 0 | 0 | 2 | 1 | 0 | **5** |
| Homoserine lactone | 0 | 0 | | 0 | 0 | 0 | 0 | 1 | 0 | 0 | **1** |
| Indole | 0 | 0 | | 2 | 0 | 1 | 0 | 1 | 1 | 1 | **6** |
| Ladderane | 0 | 1 | | 0 | 0 | 0 | 0 | 3 | 0 | 0 | **4** |
| Lanthipeptide | 1 | 1 | | 4 | 3 | 0 | 3 | 2 | 2 | 1 | **17** |
| Linear azol(in)e-containing peptide | 2 | 0 | | 1 | 0 | 0 | 1 | 0 | 0 | 0 | **4** |
| Lasso peptide | 0 | 3 | | 1 | 0 | 1 | 0 | 0 | 0 | 0 | **5** |
| Melanin | 0 | 2 | | 2 | 1 | 2 | 1 | 0 | 1 | 1 | **10** |
| Non-ribosomal peptide synthetase | 5 | 7 | | 12 | 4 | 2 | 7 | 10 | 4 | 4 | **55** |
| Non-ribosomal peptide synthetase-like fragment | 2 | 1 | | 4 | 1 | 1 | 0 | 5 | 1 | 2 | **17** |
| Nucleoside | 0 | 0 | | 1 | 0 | 0 | 0 | 0 | 0 | 0 | **1** |
| Uncategorized | 0 | 0 | | 0 | 1 | 1 | 2 | 0 | 1 | 3 | **8** |
| Phosphoglycolipid | 0 | 0 | | 1 | 0 | 0 | 0 | 0 | 0 | 0 | **1** |
| Other types of PKS | 1 | 4 | | 1 | 1 | 0 | 2 | 1 | 0 | 0 | **10** |
| Siderophore | 2 | 4 | | 3 | 3 | 3 | 2 | 3 | 3 | 3 | **26** |
| Type I PKS (Polyketide synthase) | 2 | 9 | | 6 | 4 | 0 | 7 | 14 | 3 | 1 | **46** |
| Type II PKS | 2 | 2 | | 1 | 2 | 1 | 1 | 1 | 2 | 2 | **14** |
| Type III PKS | 3 | 1 | | 1 | 3 | 2 | 0 | 0 | 3 | 1 | **14** |
| Terpene | 6 | 7 | | 12 | 5 | 3 | 4 | 10 | 7 | 6 | **60** |
| TfuA-related RiPPs | 0 | 0 | | 0 | 0 | 1 | 0 | 0 | 0 | 0 | **1** |
| Thiopeptide | 1 | 0 | | 0 | 0 | 0 | 0 | 0 | 0 | 0 | **1** |
| Trans-AT PKS fragment no trans-AT domain | 0 | 0 | | 0 | 0 | 0 | 1 | 0 | 0 | 0 | **1** |
| Total | **35** | **49** | | **63** | **33** | **25** | **37** | **65** | **33** | **29** | **369** |

* Biosynthetic gene clusters (BGCs) were predicted using the online antiSMASH web server (antismash.secondarymetabolites.org).

**Supplemental Table S4.** **Total number of VOCs from different chemical classes identified.**

| Chemical Class | *S. aureofaciens* | *S. avermitilis* | *S. clavuligerus* | *S. coelicolor* | | *S. exfoliatus* | *S. griseofuscus* | *S. hygroscopicus* | *S. lividans* | *S. parvulus* |
| --- | --- | --- | --- | --- | --- | --- | --- | --- | --- | --- |
| Alcohol | 2 | 8 | 8 | 1 | 5 | | 8 | 1 | 7 | 5 |
| Aldehyde | 2 | 3 | 5 | 1 | 3 | | 1 | 2 | 1 | 4 |
| Hydrocarbons | 7 | 11 | 10 | 6 | 21 | | 9 | 11 | 10 | 15 |
| Ester | 4 | 7 | 12 | 1 | 7 | | 2 | 4 | 2 | 9 |
| Ketone | 3 | 5 | 6 | 3 | 2 | | 2 | 2 | 2 | 5 |
| Phenol | 0 | 1 | 8 | 0 | 1 | | 0 | 2 | 0 | 2 |
| Terpene | 1 | 2 | 2 | 1 | 5 | | 2 | 15 | 2 | 4 |
| Other | 0 | 6 | 1 | 1 | 4 | | 0 | 8 | 0 | 10 |
| Total identified VOCs * | **19** | **43** | **52** | **14** | **48** | | **24** | **45** | **24** | **54** |
| Peaks detected by GC-MS ^ψ^ | **202** | **207** | **230** | **180** | **172** | | **155** | **205** | **180** | **213** |

* Number of compounds originating from the media and bacteria that could be assigned identities in the current study.

^ψ^  Total number of peaks originating from the media and bacteria that were detected in the current study, but not all could be assigned identities.

**Supplemental Table S5.** **Numbers of** ***Streptomyces* specific VOCs from different chemical classes identified.**

| Chemical Class | *S. aureofaciens* | *S. avermitilis* | *S. clavuligerus* | *S. coelicolor* | *S. exfoliatus* | *S. griseofuscus* | *S. hygroscopicus* | *S. lividans* | *S. parvulus* |
| --- | --- | --- | --- | --- | --- | --- | --- | --- | --- |
| Alcohol | 0 | 2 | 2 | 0 | 1 | 1 | 0 | 1 | 0 |
| Aldehyde | 0 | 0 | 0 | 0 | 0 | 0 | 0 | 0 | 0 |
| Hydrocarbons | 0 | 0 | 0 | 0 | 0 | 0 | 0 | 0 | 3 |
| Ester | 1 | 0 | 2 | 1 | 0 | 0 | 0 | 0 | 1 |
| Ketone | 0 | 0 | 0 | 0 | 0 | 1 | 0 | 1 | 1 |
| Phenol | 0 | 0 | 0 | 0 | 0 | 0 | 0 | 0 | 0 |
| Terpene | 1 | 1 | 0 | 1 | 5 | 2 | 11 | 2 | 3 |
| Other | 0 | 1 | 1 | 0 | 0 | 0 | 4 | 0 | 3 |
| *Streptomyces* specific VOCs identified * | **2** | **4** | **5** | **2** | **6** | **4** | **15** | **4** | **11** |

* Number of compounds originating from bacteria that could be assigned identities in the current study.
